# A modified double injection model of cisterna magna for the study of delayed cerebral vasospasm following subarachnoid hemorrhage in rats

**DOI:** 10.1186/2040-7378-4-23

**Published:** 2012-11-29

**Authors:** Furat Raslan, Christiane Albert-Weißenberger, Thomas Westermaier, Saker Saker, Christoph Kleinschnitz, Jin-Yul Lee

**Affiliations:** 1 Department of Neurosurgery, University of Würzburg, Würzburg, Germany; 2 Department of Neurology, University of Würzburg, Würzburg, Germany; 3 Division of Ophthalmology & Visual Sciences, University of Nottingham, Nottingham, United Kingdom; 4 Universitätsklinikum Würzburg, Department of Neurosurgery, Josef-Schneider-Str. 11, Würzburg, D-97080, Germany

**Keywords:** Cerebral vasospasm, Cisterna magna, Double hemorrhage model, Rat, Subarachnoid hemorrhage

## Abstract

Delayed cerebral vasospasm following subarachnoid hemorrhage (SAH) is a serious medical complication, characterized by constriction of cerebral arteries leading to varying degrees of cerebral ischemia. Numerous clinical and experimental studies have been performed in the last decades; however, the pathophysiologic mechanism of cerebral vasospasm after SAH still remains unclear. Among a variety of experimental SAH models, the double hemorrhage rat model involving direct injection of autologous arterial blood into the cisterna magna has been used most frequently for the study of delayed cerebral vasospasm following SAH in last years. Despite the simplicity of the technique, the second blood injection into the cisterna magna may result in brainstem injury leading to high mortality. Therefore, a modified double hemorrhage model of cisterna magna has been developed in rat recently. We describe here step by step the surgical technique to induce double SAH and compare the degree of vasospasm with other cisterna magna rat models using histological assessment of the diameter and cross-sectional area of the basilar artery.

## Introduction

Delayed cerebral vasospasm is still one of the leading causes of morbidity and mortality in patients suffering from SAH [1,2]. For the development of preventive or therapeutic strategies, intensive research efforts deploying numerous experimental SAH models have been done in the last decades. In 1968, Brawley first described a biphasic cerebral vasoconstriction with an acute and late response following SAH in dogs [3]. Subsequently, different SAH models have been developed in various animal species. However, due to animal rights and cost issues, rats have been increasingly used for the study of vasospasm following SAH in last years [4-7]. The commonly used techniques to induce SAH in rats include: 1) endovascular perforation of the internal carotid artery; and 2) injection of autologous blood into the cisterna magna or prechiasmatic cistern. Endovascular perforation of internal carotid artery causes acute pathophysiological changes resembling most closely aneurysmal rupture in humans [4-7]. However, due to the high mortality rate in the first 24 hours neither the endovascular perforation nor the prechiasmatic injection models are well suitable to study the delayed cerebral vasospasm following SAH. Therefore, the cisterna magna model has been used frequently. The great advantages of this latter model are its simplicity and low mortality rate [8]. Based on the observation that the severity of cerebral vasospasm is exponentially related to the subarachnoid blood volume [9], several studies using double hemorrhage injection model of cisterna magna have recently been performed. The second blood injection 24 or 48 h after the first SAH induction, however, might easily injure the brainstem resulting in high morbidity and mortality. To avoid this pitfall, a modified double hemorrhage rat model employing a catheter has been developed [8]. Here the modified SAH model is described in detail and compared with other double hemorrhage rat models.

## Material

### Experimental equipment

1. Isoflurane (2-chloro-2-(difluoromethoxy)-1,1,1-trifluoro-ethane)

2. Animal respirator

3. Cotton-tipped applicators, 3–0 Silk sutures

4. Thermostatically regulated, feedback-controlled heating lamp

5. Stereotaxic device (TSE Systems GmbH, Bad Homburg)

### Setup of surgery

1. Scalpel (FeatherDisposible Scalpel, Feather Safety Razor Co., LTD, Osaka, Japan)

2. Scissors (Fine Science Tools Inc., Foster City, CA)

3. Splinter forceps (Aesculap AG, Tuttlingen, Germany)

4. Needle holder (e.g., Halsey Micro Needle Holder, Fine Science Tools)

5. Razor blade (e.g., SIH1 razor blades, Hartenstein Laborbedarf, Würzburg, Germany)

6. Phosphate-buffered saline (PBS)

7. ImageJ software (National Institutes of Health, USA) for planimetric calculation of basilar artery diameters

8. Paraformaldehyde (4%), 0.9% NaCl

9. 2-channel laser Doppler flowmeter (LDF, MBF3D; Moor Instruments) for cerebral blood flow (CBF) monitoring

10. Monitoring apparatus for continuous measurement of mean arterial blood pressure (MABP)

### Animals

Male Sprague–Dawley rats (280–350 g) were used. SAH was induced in 10 animals by injection of autologous arterial blood into the cisterna magna as described below. Five animals served as control. All protocols were approved by the regulatory authorities for animal care and use in Lower Franconia, Germany.

## Methods

### Anesthesia

General anesthesia was induced with 4% isoflurane followed by oral intubation and subsequent mechanical ventilation with an air/oxygen mixture. After intubation, isoflurane was reduced to 2.5 - 3% to maintain normal arterial blood pressure between 80–120 mmHg and blood gases throughout the surgical procedure. Body temperature was maintained at 37°C using thermostatically controlled heating lamp.

### Surgery

**Figure 1:**
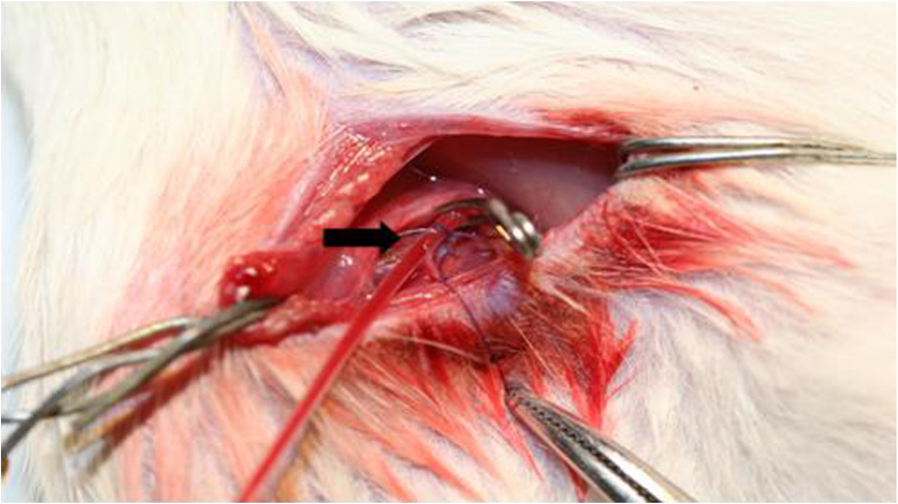
**In supine position of the rat**, **2 cm incision was performed along the groin on the right side.** The femoral artery was prepared and a PE-50 tubing was inserted into the artery (*arrow*) for continuous measurement of MABP and blood gas analysis.

**Figure 2:**
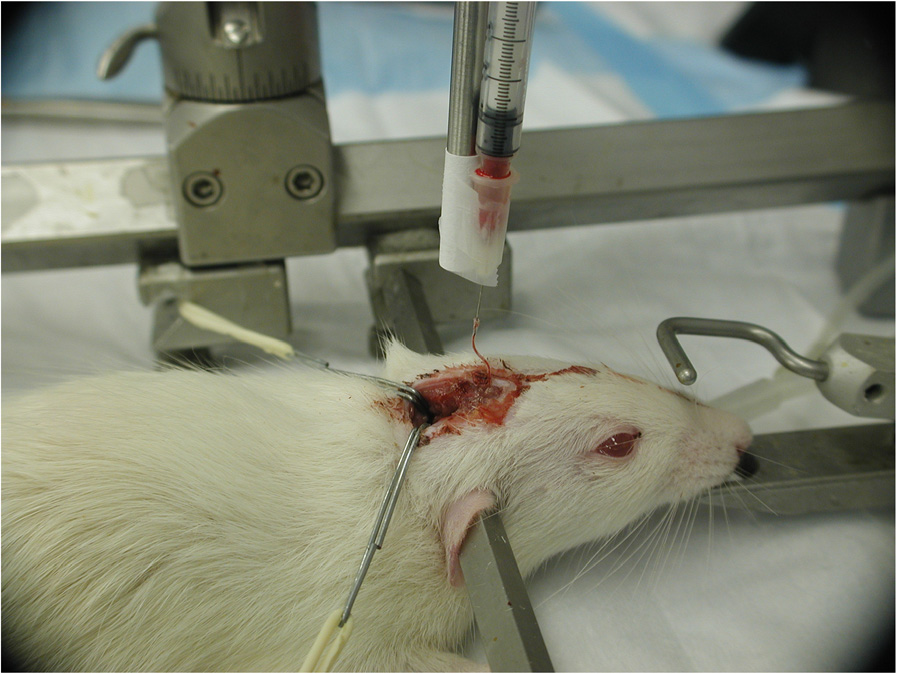
**3-point rigid cranial fixation of the head in a stereotaxic frame.** Autologous arterial blood was injected through the PE-10 catheter.

**Figure 3:**
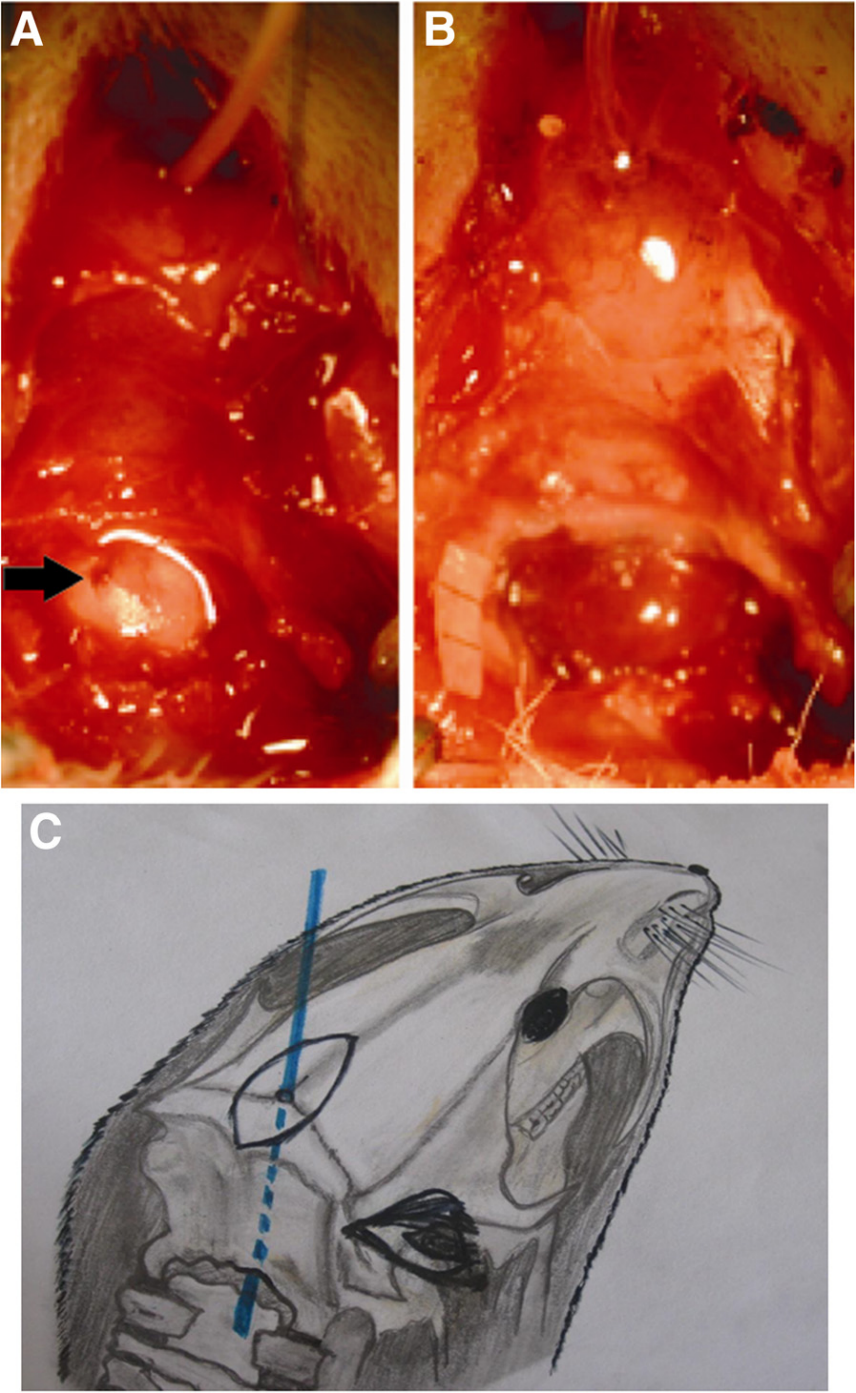
**A: After drilling a burr hole in the midline at parietooccipital suture, a PE-10 catheter was advanced into the cisterna magna (arrow).B**: Cisterna magna after injection of 0.2 ml autologous arterial blood. **C**: PE-10 catheter was advanced into the cisterna magna (blue line).

1. In supine position of the rat, a vertical skin incision (ca. 2 cm long) was made along the groin on the right side (Figure 1).

2. The femoral artery was prepared and a PE-50 tubing was introduced into the artery for continuous measurement of MABP and blood gas analysis and the fixation of the arterial tube was done to prevent dislocation. The arterial blood gas was checked immediately before SAH induction.

3. The rat was intubated with a flexible tubing and the fixation of the oral tube was done to prevent dislocation.

4. The animal was turned into a prone position.

5. Applying 3-point rigid cranial fixation in stereotaxic device as shown in Figure 2.

6. A midline scalp incision was made fronto-cervical to expose the skull including upper cervical laminae. After drilling a burr hole at bregma for placement of LDF probes, nuchal muscles were cut in the midline and stripped laterally to expose the posterior fossa, atlanto-occipital membrane and upper cervical laminae.

7. Careful resection of the lamina of atlas showing transparent dura mater and underlying cerebellum with dorsal surface of medulla oblongata.

8. Burr hole drilling in the midline just rostral to the posterior fossa bone (Figure 3A).

9. A flexible metal stylet was inserted in the lumen of PE-10 catheter, the PE-10 catheter was introduced carefully into the cranial cavity with continuous contact to the inside of the occipital bone until the catheter tip was apparent inside the cisterna magna under operating microscope, (Figure 3A).

10. Fixation of the PE-10 catheter at the skull using glue. The catheter was connected carefully to a 30 G tube clamps on the movable part of the stereotaxic device (Figure 3A).

11. Cautious aspiration of 0.1 ml cerebrospinal fluid, thereafter 0.2 ml nonheparinized blood acquired from the right femoral artery was carefully administered under visual control into the cisterna magna through the catheter during 3 min (Figure 3B). Sham-operated animals underwent through the same procedure without autologous blood injection.

12. Following the first injection, the flexible metal stylet was reinserted into the lumen of the PE-10 catheter, and then buried under nuchal muscles. Thereafter, the animal was sloping at an angle of 30° in head low position over 20 min. Sham-operated animals underwent through the same procedure without autologous blood injection.

13. Skin closure (e.g. 3–0 Ethilon suture). Isoflurane was withdrawn and the animal was allowed to wake up. The animal was transferred individually to a clean cage for recovery.

14. After 24 h, the wound was reopened, the metal stylet was removed, and 0.1 ml autologous blood was injected through the PE-10 catheter into the cisterna magna. Thereafter the PE-10 catheter was removed, and the burr hole was closed with bone wax. The animal was transferred individually to a clean cage for recovery.

**Figure 4:**
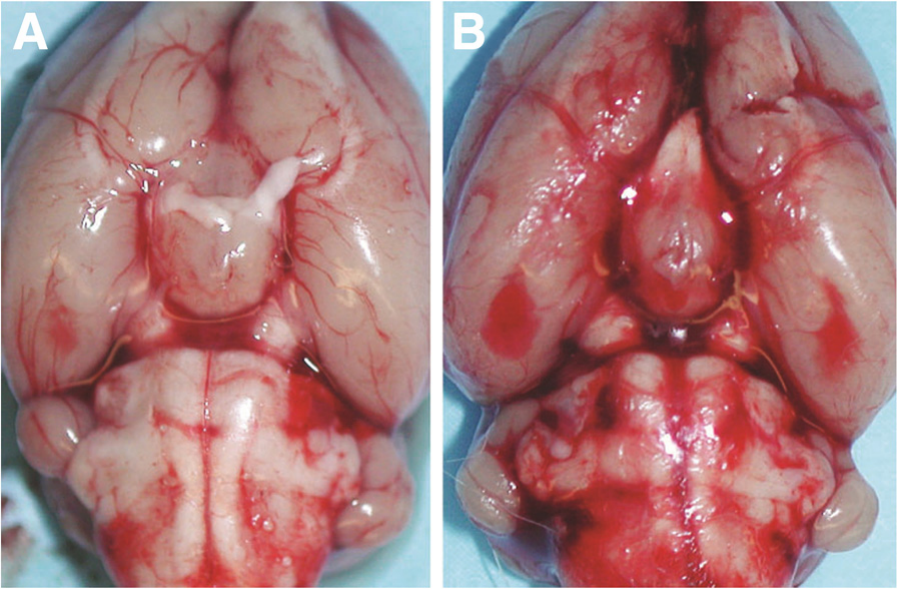
**Marked SAH was observed along the circle of Willis at the ventral brain surface (B).** Control animal (**A**).

The time required for surgery was approximately 1 hour. Five days after the second blood injection, the animals were transcardially perfused with 4% paraformaldehyde in deep anesthesia and the brain was removed for histological examination (Figure 4).

In general, the animals showed drowsiness immediately after SAH induction. In rare cases mild paresis of hind limbs could be observed. On day 1 following second SAH induction, the neurological condition rapidly improved.

### Readout parameters

Key readout parameters of the resulting vasospasm have been evaluated using a wide variety of techniques, including the assessment of neurological performance, histological examination including the measurement of the basilar artery diameter, determination of regional water content of the brain tissue, and the analysis of regional cerebral blood flow.

#### Neurological evaluation

After surgical procedure, a clinical follow-up was performed daily. Following the check of the body weight, the animals were examined with respect to alertness (e.g. drowsiness), spontaneous movements and any other neurological deficits (e.g. hemi-, para- or tetraparesis), and graded according to the method of Endo et al. (1988) as depicted in Table 1. Right hind leg weakness could be attributed to an injury of the femoral nerve [10].

**Table 1:** **Neurological grading after Endo et al**. (**1988**)

**Grade**	**Clinical findings**
1	No neurologic deficit (normal)
2	Minimum or suspicious neurologic deficit
3	Mild neurologic deficit without abnormal movement
4	Severe neurologic deficit with abnormal movement, paraplegia or quadriplegia

#### Laser doppler flowmetry

For rCBF (regional cerebral blood flow) monitoring of cerebral area supplied by middle cerebral artery, a 2-channel LDF (MBF3D; Moor Instruments, Axminster, England) was used. After the animal was placed in prone position with the head fixed in a stereotactic frame, a burr hole was drilled 5 mm lateral and 1 mm posterior to the bregma without injury to the dura mater on both sides. Then, two rectangularly bent laser Doppler probes were positioned in each burr hole using a micromanipulator. Recording was started 30 min before SAH induction and continued for 2 hours. Changes in LDF were recorded as the percentage change in initially recorded baseline values. SAH induction caused a steep decrease of rCBF to approximately 20% and 30% of the baseline following the first and second SAH induction, respectively, due to a combination of concomitant increase of intracranial pressure and early cerebral vasospasm. The reduction of rCBF recovered slowly. On day 5 after SAH induction, rCBF monitoring was repeated for 60 min as described above before animals were sacrificed showing a significant decrease of rCBF to approximately 30% of the baseline again.

#### Histological examination of the basilar artery

On day 5 after the second injection, the animals were reanesthetized, and perfusion-fixation was performed transcardially. Fixation procedure using 4% paraformaldehyde perfused via the vascular system through the heart of the rat to obtain the best possible preservation of the brain. Heart was held with forceps (it should still be beating), needle was directly inserted into protrusion of left ventricle to extend straight up about 5 mm. Needle position was secured by clamping in place near the point of entry, and the descending thoracic aorta was occluded and the right atrium opened. Procedure was performed at room temperature with 150 ml of phosphate-buffered solution (PBS, pH 7.4) and subsequently 50 ml of 4% formaldehyde. Then, the brain was immediately removed, and immersed at 4°C in 4% formaldehyde over night. For further 4 days the brain was embedded in 30% sucrose for 4 days. The brainstem was then cautious separated at the level of superior cerebellar artery (SCA) and immersed in OCT compound (Sakura Finetek USA, Inc., Torrance, CA). Thereafter, the brainstem was cut into four segments at 2 mm intervals using a rat brain slice matrix (Harvard Apparatus, Holliston, MA, USA).

**Figure 5:**
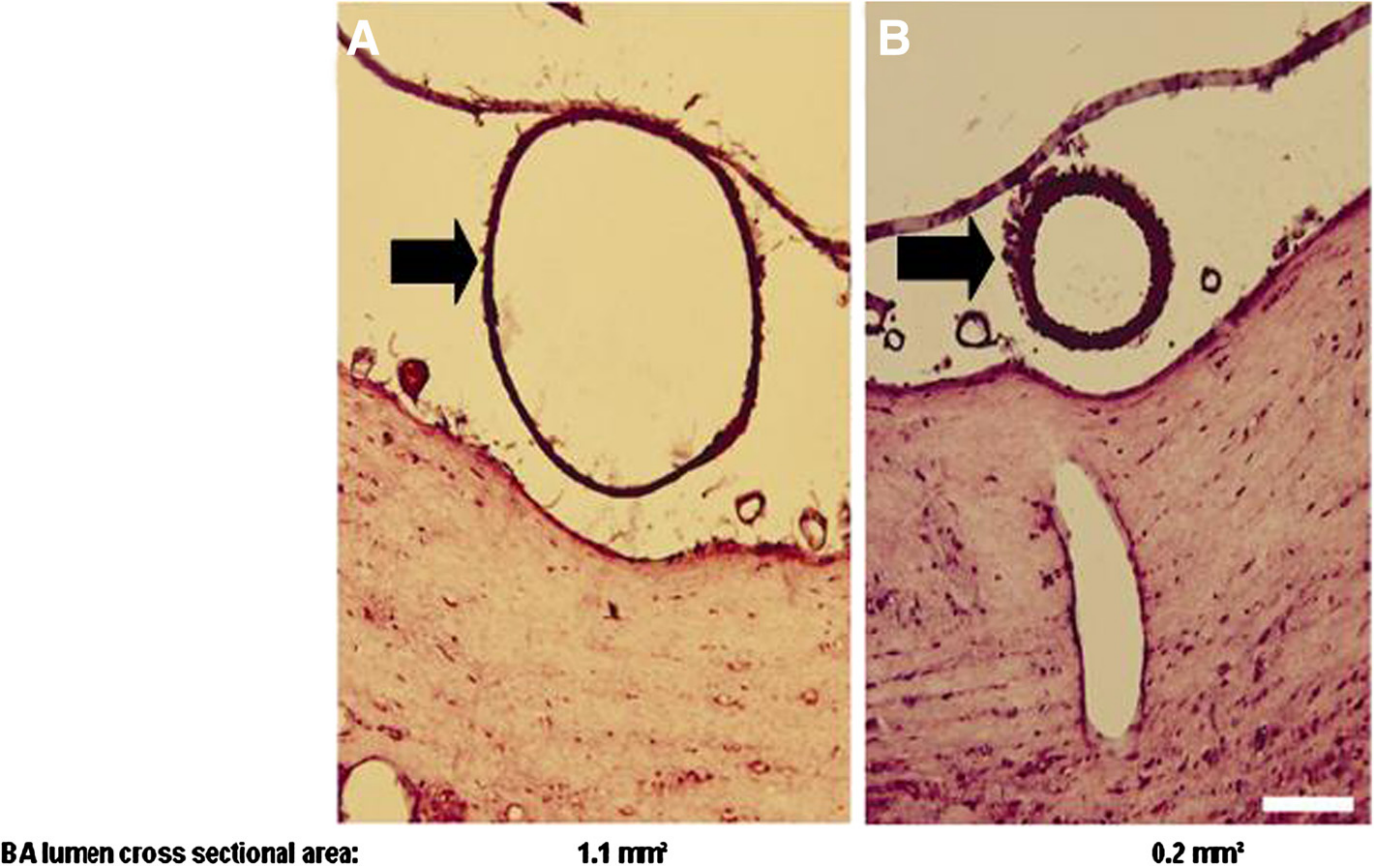
**Diameter and lumen cross sectional areas of BA 5 days after sham operation (A) and second SAH induction (B), respectively.** Marked vasoconstriction of BA was observed 5 days following second SAH induction.

At three levels of the BA 8 μm sections were cut on a cryostat: 200 μm below SCA, above and below the origin of anterior inferior cerebellar artery (AICA), and above the junction of the vertebral arteries. 10 sequential sections were cut respectively [8]. The slices were microscopically scanned. The diameter and lumen cross sectional areas of BA were determined and averaged by a blinded observer planimetrically [11] to assess the extent of vasospasm as depicted in Figure 5[12,13].

## Pearls and pitfalls

### Advantages

– The catheter-based double blood injection into the cisterna magna through a parieto-occipital burr hole causes a significant vasoconstriction of the BA leading to reduced CBF on day 5 following the second SAH induction corresponding to the delayed cerebral vasospasm following aneurysmal SAH in humans. The reduced vessel diamter of BA to approximately 60% of control was more pronounced compared to single hemorrhage cisterna magna model causing approximately 80% of control [7,8].

– The mortality rate is significantly lower compared to other double hemorrhage models involving direct puncture of the cisterna magna for blood injection using a 25- or 27-gauge sharp steel cannula leading to a high morbidity and mortality due to increased risk for brainstem lesion [14,15].

### Disadvantages

– The correct placement of the tip of PE-10 catheter is crucial. Positioning in the caudal area of cisterna magna might lead to tetraparesis following blood injection due to the compression of upper cervical medulla.

– The primary distribution of the bleeding is in the posterior fossa and not in the circle of Willis as commonly seen in the event of aneurysmal rupture in humans.

## Conclusions

The animal model of a catheter-based double blood injection into the cisterna magna through a parieto-occipital burr hole is well suitable to study the mechanism of a delayed cerebral vasospasm following SAH similar to the delayed cerebral vasospasm following aneurysmal SAH in humans. This model results in lower mortality compared to other SAH animal models, despite technical difficulties with the catheter positioning in the cranial area of cisterna magna.

## Competing interests

The authors declare no conflict of interest.

## Authors’ contributions

FR carried out the SAH experiments, performed data analysis, and drafted the manuscript. CK participated in the design and coordination of the study. TW participated in the design of the study and edited the manuscript. CAW supported FR in performing the experiments and analyzing the data. JYL initiated, designed and coordinated the study, supervised the experiments and finalized the manuscript. All authors read and approved the final manuscript.
